# The receptors for gibbon ape leukemia virus and amphotropic murine leukemia virus are not downregulated in productively infected cells

**DOI:** 10.1186/1742-4690-8-53

**Published:** 2011-07-05

**Authors:** Meihong Liu, Maribeth V Eiden

**Affiliations:** 1 Section on Molecular Virology, Laboratory of Cellular and Molecular Regulation, National Institute of Mental Health, National Institutes of Health, Bethesda, Maryland 20892, USA

## Abstract

**Background:**

Over the last several decades it has been noted, using a variety of different methods, that cells infected by a specific gammaretrovirus are resistant to infection by other retroviruses that employ the same receptor; a phenomenon termed receptor interference. Receptor masking is thought to provide an earlier means of blocking superinfection, whereas receptor down regulation is generally considered to occur in chronically infected cells.

**Results:**

We used replication-competent GFP-expressing viruses containing either an amphotropic murine leukemia virus (A-MLV) or the gibbon ape leukemia virus (GALV) envelope. We also constructed similar viruses containing fluorescence-labeled Gag proteins for the detection of viral particles. Using this repertoire of reagents together with a wide range of antibodies, we were able to determine the presence and availability of viral receptors, and detect viral envelope proteins and particles presence on the cell surface of chronically infected cells.

**Conclusions:**

A-MLV or GALV receptors remain on the surface of chronically infected cells and are detectable by respective antibodies, indicating that these receptors are not downregulated in these infected cells as previously proposed. We were also able to detect viral envelope proteins on the infected cell surface and infected cells are unable to bind soluble A-MLV or GALV envelopes indicating that receptor binding sites are masked by endogenously expressed A-MLV or GALV viral envelope. However, receptor masking does not completely prevent A-MLV or GALV superinfection.

## Background

Rubin and co-workers discovered, many years ago, that chicken embryos productively infected with Rous Sarcoma Virus (RSV) were resistant to subsequent RSV challenge [1]. This phenomenon was designated as viral superinfection interference. It was later shown that chicken embryos productively infected by RSV were resistant to avian leukosis virus [2]. It is now well established that resistance to superinfection occurs among many genera of retroviruses [3]. Cells productively infected with gammaretroviruses are resistant to challenge infection. This is thought to occur because primary viral envelope expression prevents superinfection by interfering with the binding of viruses that recognize the same receptor. It remains unclear how access of most gammaretroviruses to their receptors are blocked; in superinfection specifically, it is unclear whether the envelope protein interacts with the receptor and down modulates its expression on the cell surface or whether the receptor is masked at the cell surface by viral envelope proteins. Evidence exists for both mechanisms [4-7].

The gammaretroviruses, amphotropic murine leukemia virus (A-MLV) and gibbon ape leukemia virus (GALV), have divergent host ranges and are not in the same interference class [8]. These viruses were therefore anticipated to employ different receptors to infect target cells. When the receptors for GALV and A-MLV were cloned they were indeed shown to encode distinct but related proteins (~60% residue identity) originally designated GLVR1 and GLVR2 [8]. Later, the GALV and A-MLV receptors were identified to function as type III inorganic phosphate transporters and were renamed PiT1 and PiT2. More recently these mammalian type III sodium dependent phosphate transporters have been reclassified according to the more appropriate gene transporter nomenclature, SLC20A1 and SLC20A2, respectively [9]. SLC20A1 and SLC20A2-related proteins are present in all phyla and function as ubiquitously expressed facilitators of P_i _uptake. The SLC20A1/2 transporters permit the efficient transfer of P_i _across hydrophobic membrane barriers to provide essential nutrients required in cellular metabolism [9]. Unlike the vast majority of other carrier facilitator proteins, there are no known inhibitors of SLC20A1/2 P_i _transport [9]. Thus the effects of blocking P_i _transport by these viral receptors/type III transporters have not been directly evaluated. Surprisingly productive infection of human cells by both A-MLV and GALV is not cytotoxic. Several hypotheses could account for the absence of cytotoxic effects on cells infected by A-MLV and GALV. First, if productive infection results in receptor masking, as opposed to receptor down-regulation, the transporters on the cell surface, although their viral binding sites are no longer accessible to incoming virus, may still permit P_i _transport function as has been reported for infection with ecotropic MLV that employs the basic amino acid transporter mCAT as a receptor [10,11]. Alternately, the P_i _transporter proteins may not directly bind GALV or A-MLV but instead may function as co-receptors. This hypothesis is supported by the recent observation that GALV resistant hamster BHK cells are not rendered susceptible to GALV following the expression of SLC20A1 [12]. The ability of BHK cells, expressing SLC20A1, to bind GALV but not allow GALV entry made the role of this transporter in GALV entry more ambiguous. Finally, it is possible for cells in a culture, productively infected by both A-MLV and GALV, to remain viable despite the loss of SLC20A1/2 P_i _transport function because inorganic phosphate can be brought into infected cells by means of type II P_i _transporters or other P_i _transporters. Type II transporters normally facilitate maintaining P_i _homeostasis in the kidney and small intestine but like other genes that exhibit tissue specific expression in vivo these transporters may be turned on in cell lines in vitro making it possible for cultured cells to maintain cellular homeostasis.

To resolve the role of SLC20A1 in GALV entry and assess the effects of productive infection on SLC20A1/2, we used replication-competent A-MLV and GALV containing enhanced green fluorescence protein (eGFP) as a reporter. We also constructed GALV viruses containing fluorescence-labeled Gag proteins to observe virus-cell membrane association. These reagents, along with epitope-tagged viral receptors, allowed us to determine that both viral receptor and envelope proteins can be detected on the cell surface of productively infected cells. Finally, we showed that under receptor masking conditions, superinfection of cells productively infected with GALV can occur suggesting a mechanism of GALV entry that circumvents the SLC20A1 virus binding site.

## Results

### Superinfection resistance mediated by GALV or A-MLV

**Figure 1:**
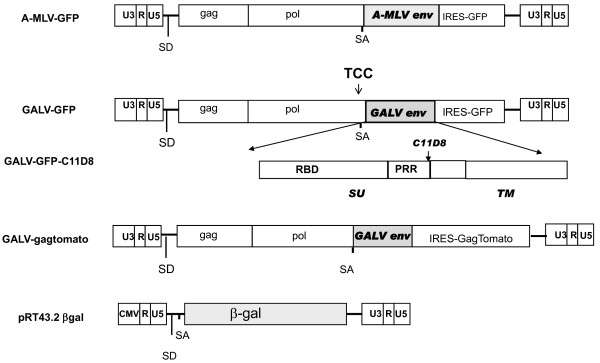
**A schematic representation of the viruses used in this study**. A-MLV-GFP and GALV-GFP are replication-competent MoMLV in which the MLV envelope (env) gene has been replaced with either A-MLV [14] or GALV env [14,15]. Both viruses contain an IRES-GFP cassette between the env gene and 3'LTR. In addition, GALV-GFP also contains an insertion of TCC just upstream of the splice acceptor (SA) resulting in a virus with enhanced infection and replication properties [15]. GALV-GFP-C11D8 is identical to GALV-GFP except that the C11D8 epitope tag (QVMTITPPQAMGPNLVLP) that derives from the amino acid terminus of the FeLV-B proline rich region (PRR) was introduced into the GALV PRR [37]. The relative position of PRR within SU and transmembrane (TM ) subunits of GALV envelope protein is shown. GALV-Gag tomato red was generated by replacing GFP of GALV-GFP with Gag fused in frame to fluorescent tomato red gene in the GALV-GFP plasmid. The retroviral vector plasmid, pRT43.2 βgal contains a CMV immediate early enhancer/promoter in the 5' LTR as well as a β-galactosidase reporter gene.

One previously employed assay to indicate receptor interference involves mixing chronically infected mink cells with viruses and demonstrating that the loss of the ability of the viruses to induce syncytia correlated with receptor interference [13]. More recently, chronically infected cells exposed to vectors expressing reporter genes have been used to assess receptor interference. The target cells that failed to express the reporter gene were considered to lack receptors due to receptor interference. In the receptor interference assays employed in the studies reported here, we used wild type A-MLV 4070A and GALV SEATO as well as replication competent pseudotyped A-MLV or GALV that had been modified to express GFP [14, 15, respectively]. These viruses previously designated AZE-GFP and MSA2-GFP by Logg et al. are schematically shown in Figure 1. AZE A-MLV-GFP or MSA2 GALV-GFP is a replication competent virus containing an MoMLV genome with either an A-MLV (AZE A-MLV-GFP) or GALV envelope gene (MSA2 GALV-GFP) substituted for that of MoMLV and as well as a GFP reporter downstream of an IRES element between envelope gene and 3' LTR. For clarity's sake AZE A-MLV-GFP and MSA2 GALV-GFP will be referred to as A-MLV-GFP and GALV-GFP, respectively, throughout the rest of the manuscript.

Since there are no antibodies available to recognize GALV envelope proteins, we further modified the GALV-GFP plasmid so that it contains an epitope tag, C11D8. The C11D8 epitope [16] was introduced in-frame after (the proline rich region) (PRR) of the envelope surface subunit of GALV-GFP and the C11D8 epitope tagged GALV-GFP is hereafter referred to as GALV-GFP-C11D8 (Figure 1). The inclusion of a GFP reporter downstream of an IRES element in these viruses allows us to use GFP as a read out monitor for initial A-MLV or GALV enveloped virus replication and spread.

Murine *mus dunni *fibroblast (MDTF) cells are non-permissive to GALV. This non-permissiveness is overcome by expressing the human receptor for GALV (SLC20A1). MDTF cells expressing hemagglutinin (HA) epitope-tagged SCL20A1 were exposed to either GALV-GFP-C11D8 or GALV wild type SEATO. One-week post exposure flow cytometric analysis (FACS) showed that more than 90% of the exposed cells were productively infected (data not shown). At this time point, infected cells were analyzed for resistance to superinfection by exposing them to GALV enveloped RT43.2 βgal vectors expressed β-galactosidase (βgal) as a reporter gene (schematically depicted in Figure 1). As shown in Table 1, GALV-GFP-C11D8 infection led to a significant blockage of superinfection by GALV/βgal vectors, similar to that observed following infection by GALV SEATO. The average GALV/βgal titer in GALV-GFP-C11D8 infected cells were 4.2 × 10^2 ^compared to an average titer of 2.1 × 10^6 ^on uninfected cells. This reduction in permissiveness is specific to GALV entry since GALV-GFP-C11D8 infection did not cause a reduction in susceptibility to A-MLV enveloped retroviral vectors expressing βgal (Table 1). Because MDTF cells express a functional receptor for A-MLV but not GALV, this result suggests that GALV infection renders MDTF/SLC20A1 specifically nonpermissive for GALV infection while retaining susceptibility to A-MLV via the murine SLC20A2 receptor.

**Table 1:** Superinfection resistance in cells infected with GALV or A-MLV

Cell lines	Primary virus	Challenge virus	Infection by challenge virus (no. of blue foci)^a^
MDTF SLC20A1-HA	Not infected	GALV/βgal	2.1 × 10^6^
		
		A-MLV/βgal	1.9 × 10^6^
	
	GALV-GFP-C11D	GALV/βgal	4.2 × 10^2^
		
		A-MLV/βgal	1.6 × 10^6^
	
	SEATO	GALV/βgal	3.1 × 10^2^

CHOK1 SLC20A2-HA	Not infected	A-MLV/βgal	3.4 × 10^6^
	
	A-MLV-GFP	A-MLV/βgal	5.3 × 10^2^
	
	4070	A-MLV/βgal	3.9 × 10^2^

MDBK SLC20A2-HA	Not infected	A-MLV/βgal	1.1 × 10^5^
		
		GALV/βgal	3.6 × 10^5^
	
	A-MLV-GFP	A-MLV/βgal	<10
		
		GALV/βgal	3.2 × 10^5^

^a^The number of blue foci observed in cells in productively infected cells 48 hours after exposure retroviral vectors containing the lacZ gene (see Materials and Methods). This number represents the average titer obtained from two independent experiments.

To assess the specific affects of A-MLV infection on challenge infection by A-MLV vectors, CHOK1 cells were used. CHOK1 cells are non-permissive to A-MLV. CHOK1 cells expressing SLC20A2, exposed to A-MLV-GFP and wild type A-MLV 4070 at one week post-infection, were challenged with A-MLV envelope vectors expressing βgal. Challenge infection was significantly reduced in A-MLV-GFP and A-MLV 4070 infected cells (Table 1). Cells productively infected with A-MLV showed resistance to challenge infection by vectors bearing A-MLV envelope similar to that observed with GALV in cells productively infected by GALV (Table 1).

Finally, to demonstrate the specificity of receptor masking, we infected bovine MDBK cells expressing SLC20A2-HA with A-MLV-GFP. MDBK cells are susceptible to GALV but not A-MLV. MDBK cells expressing SLC20A2 are susceptible to A-MLV. MDBK cells expressing SLC20A2-HA were exposed to A-MLV-GFP and one month later exposed to either A-MLV/βgal or GALV/βgal vectors. As reported in Table 1, A-MLV infection renders MDTF/SLC20A2-HA cells resistant to A-MLV/βgal but not GALV/βgal vectors.

### Viral receptors are masked but not downregulated on GALV and A-MLV infected cells

**Figure 2:**
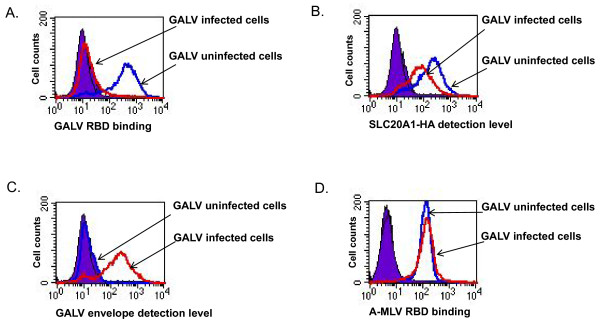
**Representative flow cytometric analyses carried out on control uninfected and GALV-GFP-C11D8 infected MDTF cells expressing the HA-tagged GALV receptor SLC20A1 cells**. The cells were stained with monoclonal antibodies against V5, HA and C11D8 epitopes as well as R-phyoerythrin conjugated goat anti-mouse isotope specific secondary antibodies. In histograms, solid purple represents control groups; blue lines represent uninfected MDTFSLC20A1-HA cells; red lines represent MDTFSLC20A1-HA cells infected with GALV-GFP-C11D8 viruses. The relative amounts of cell surface detected V5-tagged GALV RBD (A), HA-tagged SLC20A1 (B) GALV envelope tagged with C11D8 epitope (C) and V5-tagged A-MLV RBD (D) are shown on the x-axis. In these experiments, we employed MDTF or CHOK1 cells as negative controls (data not shown). The experiment was performed for three independent times with similar results.

To investigate the mechanism underlying resistance to GALV superinfection, we assayed MDTF cells expressing the GALV receptor productively infected with GALV-GFP-C11D8 and performed three FACS-based experiments. In the first assay, we assessed the ability of GALV envelope proteins to bind GALV infected cells. The second assay employed was used to detect the surface expression levels of the GALV receptor (SLC20A1) in infected cells. The third assay used was to detect the presence of C11D8 epitope tagged GALV envelope on the surface of GALV infected cells. As shown in Figure 2A, binding of V5-epitope tagged soluble GALV envelope was blocked in MDTFSLC20A1-HA cells productively infected with GALV for one week. However, the GALV receptor level was only modestly downregulated compared to uninfected cells (Figure 2B). GALV envelope proteins are expressed and present on the surface of cells productively infected with GALV-GFP-C11D8 (Figure 2C). To show that the blocking of binding is specific, we examined the ability of soluble A-MLV RBD (the receptor binding domain of the envelope protein) to bind to GALV infected MDTF cells expressing SLC20A1. As shown in Figure 2D, GALV infection blocked GALV RBD but not A-MLV RBD binding, indicating that GALV infection specifically restricts the ability of GALV RBD to bind GALV infected cells.

**Figure 3:**
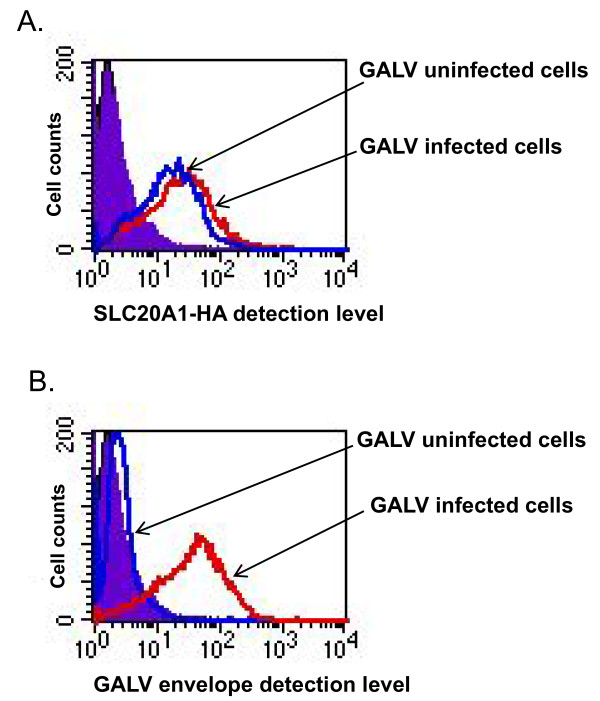
**FACS analysis of SLC20A1-HA expression and GALV (C11D8) envelope associated with the surface of MDTFSLC20A1-HA cells chronically infected (one month-post exposure) with GALV-GFP-C11D8 is shown in histograms**. The level of SLC20A1-HA expression (A) and the relative amount of GALV envelope glycoprotein (C11D8) bound to the cells (B) on the surface of MDTFSLC20A1-HA cells uninfected or chronically infected with GALV-GFP-C11D8 viruses. We employed MDTF cells as negative controls for receptor detection and viral infection (data not shown). The experiment was performed three independent times, and images are from one representative experiment.

To investigate whether SLC20A1 is down-regulated in cells chronically infected with GALV (e.g., greater than one month post exposure) GALV-GFP-C11D8 infected cells, we again performed the same three assays used for the assessment of A-MLV and obtained similar results (Figure 3). MDTFSLC20A1-HA cells chronically infected with GALV expressed both the GALV receptor (SLC20A1-HA) and GALV envelope proteins on the surface of infected cells.

**Figure 4:**
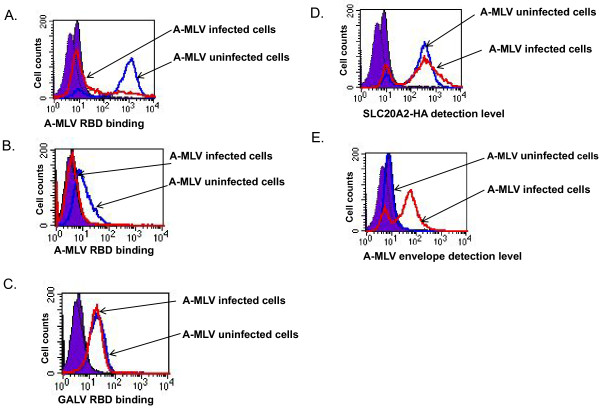
**SLC20A2-HA expression, A-MLV envelope and soluble A-MLV or GALV RBD bound to the surface of CHOK1 cells expressing SLC20A2-HA or MDBK cells expressing SLC20A1-HA cells chronically infected with A-MLV-GFP (one month after infection) or uninfected control cells was assayed by FACS and displayed in histograms**. The cells were stained with primary antibodies specifically against A-MLV (83A25) and HA and V5 epitopes. The corresponding secondary antibodies used are species and isotope specific and conjugated with R-phyoerythrin. Solid purple lines represent control groups; blue lines represent uninfected cells; red lines represent cells infected with A-MLV-GFP. V5 epitope tagged A-MLV RBD bound to CHOK1 expressing SLC20A2-HA cells (A) or to MDBK expressing SLC20A2-HA cells (B) and V5 epitope tagged GALV RBD bound to MDBK cells expressing SLC20A2-HA (C), The expression level of HA-tagged SLC20A2 on the surface of CHOK1 expressing SLC20A2-HA cells (D), or the relative amounts of A-MLV envelope bound to CHOK1 expressing SLC20A2-HA cells (E) are shown on the x-axis. The experiment was performed three times, and images are from one representative experiment.

Similar assays were undertaken with cells infected with A-MLV. As mentioned above, hamster CHOK1 are resistant to A-MLV, but are rendered susceptible after expressing SLC20A2-HA, a HA-epitope tagged form of the human receptor for this virus. A-MLV receptors were detected on the surface of CHOK1SLC20A2-HA cells productively infected with A-MLV (one month after initial viral exposure) at a level similar to that observed on uninfected cells (Figure 4D). The presence of A-MLV envelope proteins on the surface of A-MLV infected cells was detected using the 83A25 rat monoclonal antibody [17] (Figure 4E). A-MLV infected CHOK1SLC20A2-HA did not bind V5-tagged A-MLV RBD (Figure 4A). In addition, A-MLV RBD binding (Figure 4B) but not GALV RBD binding (Figure 4C) was blocked in A-MLV infected MDBK cells expressing SLC20A2-HA, indicating that the block to binding is virus specific.

In Table 2, we summarize the results obtained with the cell lines (MDTF and CHOK1 cells expressing different receptors) and viruses (wild type A-MLV 4070A and GALV SEATO as well as the chimeric replication competent A-MLV-GFP, and GALV-GFP) assessed in this study. Altogether, our results suggest that receptor masking is the major mechanism for GALV and A-MLV superinfection resistance. It is also possible that the inability of envelope RBD to bind to cells productively infected with the appropriate virus is mediated by an indirect mechanism and not by direct binding of endogenously produced envelope to virus receptor. To determine whether endogenous envelope expressed in cells productively infected with GALV is physically associated with SLC20A1 proteins we performed co-immunoprecipitation assays and crosslinking experiments.

**Table 2:** Detection of the receptors and viral envelope proteins present on the surface of cells chronically infected with GALV or A-MLV viruses over one month.

	Primary infection	Receptor present on the cell surface	Viral envelope present on the cell surface
MDTFSLC20A1-HA	GALV-GFP-C11D8	Yes^a^	Yes

MDTFSLC20A1-HA	SEATO	Yes	ND^b^

CHOK1SLC20A1-HA	GALV-GFP-C11D8	Yes	Yes

CHOK1SLC20A1-HA	SEATO	Yes	ND

CHOK1SLC20A2-HA	A-MLV-GFP	Yes	Yes

CHOK1SLC20A2-HA	4070	Yes	Yes

MDTFSLC20A2-HA	A-MLV-GFP	Yes	Yes

MDTFSLC20A2-HA	4070	Yes	Yes

^a ^Yes means the ability to detect viral receptor or envelope protein on the surface of cells determined by FACs.)

^b ^ND means not determined

### GALV envelope proteins physically associate with SLC20A1

**Figure 5:**
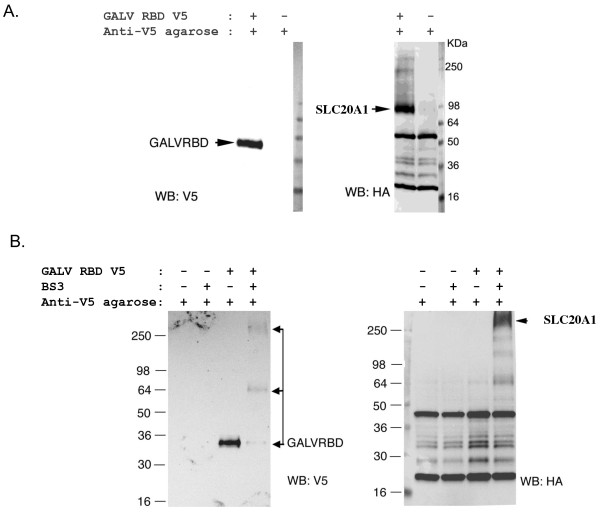
**SLC20A1 protein physically interacts with GALV envelope protein. MDTFSLC20A1-HA cells incubated with V5 tagged GALV RBD were lysed, co-immunoprecipitated with agarose beads covalently linked to V5 antibody and subjected to western blots probed with antibody to V5 or HA** (A). Western blots of the cells cross-linked with BS3 and then immunoprecipitated with agarose beads covalently linked to V5 antibody and subjected to western blot analysis using antibody to V5 or HA as a probe (B).

Even though SLC20A1 has been demonstrated to facilitate GALV entry into murine cells, a direct physical association of GALV envelope protein with SLC20A1 has not been shown. To provide experimental support for receptor masking is a result of the direct association of GALV envelope and its receptor SLC20A1 we performed co-immunoprecipitation (coIP) and cross-linking coIP assays to assess whether GALV envelope protein and SLC20A1 directly interact. For coIP assays, after MDTFSLC20A1-HA cells were incubated with V5-tagged GALV RBD, a crude cell membrane preparation was made from the cells and the V5-tagged GALV RBD protein and its associated proteins in a crude cell membrane preparation were then precipitated by the addition of sepharose beads covalently coupled to anti-V5 monoclonal antibody. The proteins bound to the beads were then eluted by the addition of SDS-loading buffer and analyzed by western blot (Figure 5A). Bis (sulfosuccinimidyl) substrates (BS3), a reagent commonly employed to crosslink cell-surface proteins and identify receptor-ligand interactions was used to further validate the association of SLC20A1-HA and GALV RBD-V5. MDTFSLC20A1-HA cells in suspension were exposed to GALVRBD-V5 and then incubated with BS3. Cell membrane lysates were prepared and V5-tagged GALV RBD and its associated proteins crosslinked by BS3 in the cell membrane lysates were then precipitated by the addition of beads coupled to anti-V5 monoclonal antibody. As shown in Figure 5B, an immunoprecipitated complex larger than 250Kda was detected with an antibody to HA (blot on right, Figure 5B). Another blot was probed with a V5 antibody (blot on left, Figure 5A). The results shown in these Western blots suggest that GALV RBD and SLC20A1 are part of the BS3 crosslinked complex that can be pulled down by anti-V5 antibody. Together, the results shown in Figure 5 indicate that GALV directly interacts with SLC20A1. Therefore, it is reasonable to posit that the GALV envelope protein present on the surface of infected cells remains associated and occupies the viral binding site on SLC20A1 thus preventing GALV superinfection or the binding of soluble GALV RBD to infected cells.

### GALV superinfection occurs in productively infected cells under receptor masking conditions

**Figure 6:**
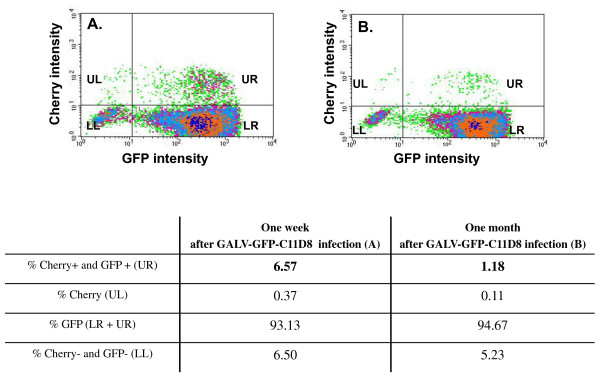
**GALV enveloped retroviral vectors expressing cherry red protein superinfecting MDTFSLC20A1-HA cells productively infected with GALV (GALV-GFP-C11D8) for one week (A) or one month (B)**. After 48 hours, the cells were harvested for FACs analysis and the densitograms (top panel) and the quadrant statistics are presented in the table at the bottom. The experiment was performed three independent times, and the representative analysis is presented.

With advanced live image technology, cells productively infected with the ecotropic retrovirus, Moloney murine leukemia virus (MoMLV), have been shown to have MoMLV particles surfacing on their cell membranes. These particles move inward towards the cell body of chronically infected cells, in vitro, when polybrene is added to the media [18]. This previous report suggests that, under certain conditions, superinfection of productively infected cells can occur at least for MoMLV. As shown in Table 1 we found that superinfection can occur when GALV/A-MLV infected cells are exposed to GALV/βgal or A-MLV/βgal vectors, albeit inefficiently. Therefore, we next attempted to determine whether low-level re-infection occurs in cells that have already been productively infected, that is, under conditions of receptor masking. We exposed MDTFSLC20A1-HA to GALV-GFP-C11D8 and continuously cultured them for one week and one month; then, we individually exposed them to GALV-enveloped vectors, expressing cherry red fluorescent protein (GALV/cherry) as an indicator of infection. After 48 hours, these cells were analyzed by flow cytometry. Of the 93.13% MDTFSLC20A1-HA cells productively infected with GALV-GFP-C11D8 for one week, only 6.57% were susceptible to superinfection with GALV-enveloped retroviral vectors expressing cherry red protein (Figure 6A). The continual culture of GALV-GFP-C11D8 infected MDTFSLC20A1-HA cells for one month resulted in a decrease in infection to 1.18% compared to 94.67% of the initially infected cells (Figure 6B).

**Figure 7:**
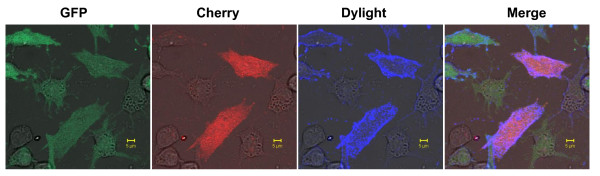
**Immunofluorescence confocal microscopy of superinfection of GALV infected cells**. MDTFSLC20A1-HA cells infected with GALV-GFP-C11D8 for one month and then exposed to GALV enveloped vector expressing cherry red fluorescent protein. After 48 hours, cells were fixed and stained by C11D8 monoclonal antibody and dylight conjugated goat anti-mouse IgG antibody.

The superinfected cells were also examined for surface expression of GALV envelope protein bearing a C11D8 epitope using immunofluorescence confocal microscope, C11D8 monoclonal antibody and a dylight conjugated anti-mouse IgG (blue fluorescence). A small number of cells productively infected with GALV-GFP-C11D8 (GFP positive) were also susceptible to GALV/cherry vectors (cherry positive). These superinfected cells (GFP positive and cherry positive) also expressed GALV envelope on their surface as detected by C11D8 Dylight (blue) staining (Figure 7). We employed three controls in these assays (1) uninfected MDTFSLC20A1-HA cells (negative for GFP, cherry red expression and C11D8 Dylight staining) (2) MDTFSLC20A1-HA cells exposed to GALV/cherry vectors (negative for GFP expression and C11D8 Dylight staining) and (3) MDTFSLC20A1-HA cells infected with GALV-GFP-C11D8 over one month (negative for cherry red expression) (data not shown). These results indicate that superinfection can occur in cells productively infected with GALV under conditions of receptor masking.

### GALV particles efficiently attach to infected cell surface

**Figure 8:**
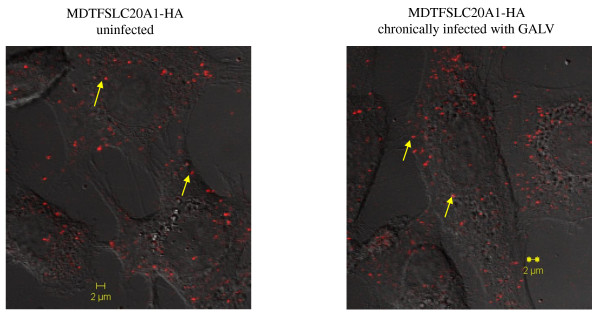
**Nonspecific attachment of GALV to chronically infected MDTFSLC20A1-HA cells**. MDTFSLC20A1-HA cells uninfected or chronically infected with GALV were adsorbed with fluorescently labeled GALV viruses (GALV-Gag tomato red). Images were taken at 63x magnification on a LSM510 invert Meta confocal microscope. The arrows point to the tomato labeled GALV particles. The images are representatives of three independent experiments.

Previously, it has been reported that MLV viruses can nonspecifically bind to target cells and the binding is receptor-independent [19-21]. Therefore, we hypothesized that when GALV productively infected cells are exposed to GALV, these GALV particles may still be capable of efficiently attaching to the cells and responsible for superinfection under some conditions (e.g., in the presence of polybrene), even though the binding sites of SLC20A1 receptors are occupied by GALV envelope. To test this hypothesis, we made GALV particles containing tomato red fused to its Gag viral proteins. We modified GALV-GFP by substituting IRES-GFP with IRES-MLV gag fused with the gene encoding tomato red (schematically shown in Figure 1). Uninfected and GALV-GFP-C11D8 chronically infected (one month post initial exposure to virus) MDTFSCL20A1-HA cells were exposed to the fluorescent GALV, incubated for 1 hour at 37°C, extensively washed, fixed and then, examined using a immunofluorescence confocal microscope. We observed that the tomato red GALV particles bound to the chronically infected cells at a level similar to those bound to uninfected cells (Figure 8) by manual visual assessment of the number of the tomato red GALV particles attached to the cell surface. Between 70 and 100 particles are associated with individual infected cells. A similar number of tomato red GALV particles were determined to be cell surface associated on MDTF/SLC20A1 cells (data not shown). Thus exogenous GALV particles bind uninfected cells expressing viral receptors as efficiently as infected cells expressing occupied viral receptors.)

## Discussion

Retrovirus superinfection resistance is an important feature of productively infected cells. The inability of chronically infected cells to block superinfection is frequently associated with cytopathic effects that can result in cell death [22-25]. Two envelope-mediated mechanisms have been proposed for superinfection resistance, receptor downregulation and receptor masking [4,5,7,13,26]. In this report, we investigated one mechanism by which A-MLV and GALV mediate resistance to superinfection. We used replication- competent viruses expressing either GALV or A-MLV envelope proteins together with a GFP reporter gene, GALV-enveloped viruses expressing fluorescence-labeled Gag proteins and antibodies reactive with viral particles, their receptors or soluble envelope proteins. Using this repertoire of reagents, we clearly demonstrated that A-MLV or GALV receptors are masked by viral envelope protein. In cells productively infected with A-MLV or GALV we observed: i: both GALV SLC20A1 and A-MLV SLC20A2 receptors remain present on their respective infected cell surface (Figures 2, 3 and 4); ii: GALV and A-MLV envelope glycoproteins are detected on the surface of infected cells (Figures 2, 3 and 4); iii: infected cells are not able to bind GALV RBD or A-MLV RBD suggesting that the binding sites on these receptors are occupied by viral envelope proteins. We could not, however, rule out that down regulation of receptors also occurs in a small portion of cells chronically infected with GALV or A-MLV.

We have previously reported that A-MLV infection of NIH-3T3 cells overexpressing epsilon-epitope-tagged SLC20A2 results in the redistribution of epsilon-epitope tagged SLC20A2 inside the cell [27]. Confocal microscopy provides representative images of *permeabilized *infected cells and not quantitative analyses. It has now known that in uninfected cells, PiT2 is detectable in both the cytoplasm and on the cytoplasmic membrane of permeabilized cells based on more recent findings [28,29]. In this report we have undertaken quantitative comparisons of uninfected and A-MLV infected cells in parallel and demonstrated something not addressed in the prior confocal microscopy studies [27]. The use of FACs analysis allows several advantages over confocal microscopy i) the use of live not fixed nor permeabilized cells for cell surface receptor expression, RBD binding or viral envelope binding analyses ii) dead cells are eliminated from the analyses prior to FACS by propidium iodide staining iii) the employment of GALV or A-MLV expressing GFP in this study allows FACS gating and evaluation of infected cell populations as opposed to uninfected cells and iv) FACs analysis provides quantitative population statistics data.

In Figure 2, we show that GALV infection may be accompanied by a slight down regulation of SLC20A1. However, receptor down regulation is a minor event not a major event accompanying GALV or A-MLV infection. Our conclusions are based on studies on two types of cells lines (MDTF or CHOK1) as well as wild type viruses, GALV (SEATO) and A-MLV (4070) and replication competent modified GALV or A-MLV expressing GFP.

The knock-out of SCL20A1 in mice has been reported to result in embryonic lethality. Furthermore, in vitro depletion of SLC20A1 in cell lines impairs their cellular proliferation [30]. The reasons why deleterious events were not evident in cells infected with GALV may be accounted for in a number of ways. First, receptor masking does not completely abolish transporter function. This is supported by the report that in mouse fibroblasts expressing A-MLV envelope glycoprotein, only a partial reduction of P_i _transport is observed [31]. Furthermore, chronic infection with another gammaretrovirus E-MLV that uses a basic amino acid transporter as a receptor causes only a 50-70% loss of transporter function in plasma membranes [11]. Secondly, Bottger et al. have reported that the residues important in sodium phosphate symporter function [32] are outside of the regions implicated as the receptor binding sites for these proteins [33,34]. Finally the ability to directly assess SLC20-mediated P_i _transport under conditions of productive infection in infected cell lines in culture is compromised by the tendency of cultured cells to express a broader range of genes than those expressed in the tissues they derive from. In the case of MDTF cells, this could extend to the expression of the kidney specific type II P_i _transporters.

We also tried to resolve the mechanism by which the rare incidence of superinfection occurs in a chronically infected population of cells. Specifically is the "superinfection event" occurring in a cell that is either not infected, infected but no longer expressing viral envelope proteins (i.e., a situation analogous to viral latency), or productively infected and expressing envelope proteins. We used GALV replication competent viruses containing GFP as a reporter for primary infection and GALV enveloped vectors expressing cherry red to determine superinfection capability. Approximately 95% of the cells were infected by GALV-GFP one month after initial exposure (Figure 6) at which point these cells were demonstrated to be weakly susceptible (1%) to superinfection by vectors expressing cherry red. Superinfection of GALV cherry red vectors is evident in cells expressing both green and red fluorescent proteins (Figure 6 and 7). Superinfected cells also express GALV envelope proteins on their cell surface (Figure 7) suggesting that they are productively infected with GALV and maintain viral envelope expression on the infected cells surface. Therefore, approximately 6.57% or 1.18% superinfection occurs in productively infected cells and not in the 6.50% or 5.23% cells that are not infected by GALV-GFP or in the productively infected cells that retain only GFP gene expression but not viral gene expression (Figure 7). Nonspecific absorption of MLV has been described for a variety of cell types [19-21]. We observed adsorption of infectious Tomato red GALV viral particles on MDTFSLC20A1 cells chronically infected with GALV-GFP (Figure 8). Therefore, it remains possible that superinfection of GALV can occur via "activation in trans" as has been previously described for facilitating the ability of bound, but noninfective virus to enter cells after being exposed to soluble envelope proteins [35]. Alternately, a few unoccupied SLC20A1 on the cell surface could permit GALV superinfection.

## Conclusion

In conclusion, resistance to GALV or A-MLV mediated superinfection occurs through receptor masking, not receptor down-regulation. Cells productively infected with either GALV or A-MLV retain the ability to bind viral particles and these bound particles may participate in the rare superinfection event that occurs in infected cells.

## Methods

### Cell Culture

Cell lines used in this study include murine Mus dunni tail fibroblasts MDTF [36], Chinese hamster ovary (CHOK1) cells, CCL61, (ATCC, Manassas, VA), human embryonic kidney 293T cells (Cell Genesys, Foster City CA) Mason-Darby bovine kidney cells (CCL 22, ATCC, Manassas, VA). All cells, with the exception of CHOK1, were maintained in Dulbecco's modified Eagle's medium with Glutamax (DMEM) (Invitrogen, CA), supplemented with 10% fetal bovine serum, 100 units of penicillin/ml, and 100 ug of streptomycin/ml. CHOK1 cells were maintained in alpha minimal essential medium (MEM) supplemented with 10% fetal bovine serum, 100 units of penicillin/ml, and 100 ug of streptomycin/ml. (Invitrogen, CA). Stable cell lines expressing the GALV viral receptor SLC20A1 or A-MLV viral receptor SLC20A2 tagged with HA were made by using vesicular stomatitis virus (VSV) G protein-enveloped vectors with genomes expressing the appropriate receptor cDNA in the retrovirus pLNSX packagable genome and selected for G418 resistance [29].

### Plasmids

A-MLV-GFP and GALV-GFP (generously provided by Christopher Logg, University of California, Los Angeles, CA,) are replication-competent E-MLVs but contain A-MLV or GALV env protein that replaces E-MLV env gene respectively, as well as an IRES cassette between the env gene and 3' untranslated region. In addition, the GALV-GFP contains an insertion of TCC just upstream of the splice acceptor to allow more efficient replication (Figure 1) [15]. GALV-GFP-C11D8 was constructed by introducing a C11D8 epitope tag at 3' of the PRR within the GALV env gene (Figure 1) [37]. GALV-Gag tomato red was constructed by replacing the IRES-GFP cassette in the GALV-GFP construct with IRES-Gag fused in frame to a fluorescent tomato gene. Briefly, we prepared the following three fragments: an IRES fragment amplified by PCR, cleaved with Mlu and EcoRI sites, a gag fragment obtained from MLV-gag-YFP plasmid (Addgene Inc.) [38] by double digestion with EcoRI and NheI and tomato red gene fragment amplified by PCR from the CMV-brainbow-1.0-L plasmid (Addgene Inc.)[39] then cleaved with NheI and Not. All three fragments were ligated to GALV-GFP plasmid after digestion with MluI and NotI (Figure 1). The sequences of all plasmids used in these analyses were verified.

### Production of replication competent retroviruses and retroviral vectors

293T cells were seeded at a density of 10^6 ^per 10 cm dish and the appropriate plasmids were introduced by calcium phosphate transfection (Promega) as previously described [29]. Soluble GALV RBD and A-MLV RBD were generated by transient transfection of 293T cells using plasmids expressing GALV envelope surface unit (SU) or A-MLV envelope SU tagged with V5 as previously described [37]. The GALV-GFP, GALV-GFP-C11D8-, GALV-GagTomato red or A-MLV-GFP plasmids were transfected into 293T cells to make GALV or A-MLV pseudotyped replication-competent viruses. Supernatants containing GALV or A-MLV enveloped retroviral vectors, soluble GALV or A-MLV RBD or replication-competent viruses were harvested 48 to 72 h post-transfection, then passed through a 0.45 mM Millipore (Bedford, Mass.). Supernatant containing GALV-GagTomato viruses was further concentrated using Beckman ultracentrifuge at 25,000 rpm at 4°C for 2 hours through a 25% sucrose cushion.

### Stable Cell Lines

Stable cell lines expressing the GALV receptor SLC20A1 or A-MLV receptor SLC20A2, tagged with HA, were made by exposing appropriate target cells to vesicular stomatitis virus (VSV) G protein-enveloped vector particles containing the appropriate receptor cDNA in the pLNSX genome. Transduced cells were selected for G418 resistance as previously described[29].

### Viral infection and titration

Target cells were seeded one day in advance in a 24-well plate at a density of 2 × 10^4 ^cells per well. Cells were exposed to supernatant containing respective retroviral vector and then supplemented with 10 mg/ml polybrene. Twenty-four hours later, the medium was changed and cells were cultured for an additional 36 to 48 h before analysis for expression of β-gal by histochemical staining with X-Gal (5-bromo-4 chloro-3-indolyl-b-Dgalactopyranoside), as previously described [37]. Titers were determined by averaging the numbers of blue foci (BFU) obtained with vectors for each cell line tested in three or more independent experiments and expressed as BFU/ml.

### Soluble-RBD binding assays and detection of SLC20A1 and SLC20A2 expression as well as viral envelope associated with the cell surface of infected cells

Binding assays using V5-tagged soluble A-MLV or GALV RBD were performed as previously described [29,37]. 5 × 10^5 ^target MDTF or CHOK1 cells expressing SLC20A1 or SLC20A2 receptor fused to an HA epitope tag [29] were incubated with soluble V5-tagged RBD protein at 37°C for 45 min. The cells were fixed with 1% paraformaldehyde and then analyzed by flow cytometry. HA-tagged receptors were detected on the cell surface by incubation of MDTF cells expressing receptors with monoclonal HA antibody HA.11 (Covance Inc.), followed by incubation with a secondary antibody, R-phyoerythrin-conjugated (Invitrogen, Eugene, Oregon). The detection of GALV or A-MLV envelope protein associated with the surface of cells chronically infected with respective viruses was performed by incubation of the cells with monoclonal antibody C11D8 (Santa Cruz biotechnology, Inc. CA) [14,15] or rat monoclonal antibody 83A25 (generously provided by Leonard. Evans, National Institute of Allergy and Infectious Diseases, Hamilton, Montana) [17] at room temperature for 1 hour, followed by incubation with R-phyoerythrin conjugated secondary antibody.

### Virus adsorption

MDTFSLC20A1-HA cell lines uninfected or infected over one month were grown on 35 mm tissue culture dishes with cover glass bottom of 0.17 mm in thickness overnight (World Precision Instruments, Inc.) and then incubated with 1 ml **of **20-fold concentrated GALV-Gag tomato virus at 37°C for 45 minutes. After extensive washes, cells were fixed with 2% paraformaldehyde and images were taken with LSM 510 inverted Meta confocal microscope as described below.

### Coimmunoprecipitation (Co-IP) and crosslinking Co-IP

All immunoprecipitation procedures were carried out at 4°C. 3 × 10^6 ^MDTF cells expressing HA-tagged SLC20A1 protein were incubated with 5 ml of V5 tagged GALVRBD at 37°C for 45 minutes, followed by extensive washes. Crude cell plasma membranes were then prepared as described previously [40]. Crude cell plasma membranes were transferred to tubes and centrifuged at 15,000*g *for 60 minutes. Subsequently, the membrane pellets were resuspended in 500 μl lysis buffer (150 mM NaCl, 50 mM Tris-HCl, 2.5 mM EDTA, complete proteinase inhibitor tablet and 1% CHAPs) and incubated for 30 minutes, followed by centrifugation. The resultant supernatant was incubated with anti-V5 affinity sepharose beads (Sigma) overnight. Dissociated protein complexes were then separated with 4-20% tris-glycine SDS-PAGE gel (Invitrogen, Inc. CA).

Cross linking co-IP was performed on MDTFSLC20A1-HA cells grown on 10 cm tissue culture dish by incubation with V5 tagged GALV RBD at 37°C for 45 minutes, and then treated with a homobifunctional, water-soluble, non-cleavable and membrane impermeable crosslinker, Bis(sulfosuccinimidyl)suberate (BS^3^) (Thermo Scientific Pierce) at a concentration of 1 mM in 2.5 ml HBSS buffer, on a shaker for one hour. After extensive washes with HBSS buffer, the cells were scraped off in 1 ml lysis buffer containing 1% NP40 and lysed for 20 minutes, followed by high speed centrifugation for 30 minutes. The resultant supernatant was used for crude cell membrane preparation and Co-IP as described above.

### Confocal microscopy

Cells were grown on 35 mm tissue culture dish with glass bottom of 0.17 mm in thickness over night, followed by fixation with 2% paraformadyhyde in PBS and blocked with 5% bovine serum albumin in PBS. Subsequently, cells were probed with monoclonal antibodies (C11D8) and stained with dylight conjugated goat anti-mouse IgG (Thermo scientific Pierce). Images were collected on an LSM510 invert Meta confocal microscope (Carl Zeiss, Thornwood, NY). The 488 and 568 nm lines of a krypton/argon laser were used for fluorescence excitation of GFP and cherry red respectively and 420 nm line for dylight.

### Flow cytometry

Epics XL (Beckman Coulter, Fullerton, CA) and FACScan (Becton Dickinson, Franklin Lakes, NJ) flow cytometers were used for analysis of GFP and cherry red expression in infected and transfected cells using a 525 nm or a 530 nm band pass emission filter. 20,000 cells from each sample were analyzed after trypsinization and suspension in PBS.

## Competing interests

The authors declare that they have no competing interests.

## Authors' contributions

ML helped design the study, carried out the experiments, analyzed the data, and drafted the manuscript. MVE helped design the study and write the manuscript. Both authors read and approved the final manuscript.
